# STABILON, a Novel Sequence Motif That Enhances the Expression and Accumulation of Intracellular and Secreted Proteins

**DOI:** 10.3390/ijms23158168

**Published:** 2022-07-25

**Authors:** Zsuzsanna Rethi-Nagy, Edit Abraham, Katalin Udvardy, Eva Klement, Zsuzsanna Darula, Margit Pal, Robert L. Katona, Vilmos Tubak, Tibor Pali, Zoltan Kota, Rita Sinka, Andor Udvardy, Zoltan Lipinszki

**Affiliations:** 1 Biological Research Centre, Institute of Biochemistry, MTA SZBK Lendület Laboratory of Cell Cycle Regulation, ELKH, H-6726 Szeged, Hungary; nagy.zsuzsanna@brc.hu (Z.R.-N.); abraham.edit@brc.hu (E.A.); udvardy@brc.hu (K.U.); pal.margit@med.u-szeged.hu (M.P.); 2 Doctoral School of Biology, Faculty of Science and Informatics, University of Szeged, H-6726 Szeged, Hungary; 3 Single Cell Omics Advanced Core Facility, Hungarian Centre of Excellence for Molecular Medicine (HCEMM), H-6726 Szeged, Hungary; eva.klement@hcemm.eu (E.K.); zsuzsanna.darula@hcemm.eu (Z.D.); 4 Biological Research Centre, Laboratory of Proteomics Research, ELKH, H-6726 Szeged, Hungary; 5 INPAMAC Zrt, H-6723 Szeged, Hungary; robert.katona@inpamac.com; 6 Creative Laboratory Ltd., H-6726 Szeged, Hungary; vilmos.tubak@creativelab.hu; 7 Biological Research Centre, Institute of Biophysics, ELKH, H-6726 Szeged, Hungary; tpali@brc.hu (T.P.); kota.zoltan@brc.hu (Z.K.); 8 Department of Genetics, University of Szeged, H-6726 Szeged, Hungary

**Keywords:** stabilization motif, protein degradation, protein production, secreted proteins, enhancer, mRNA stability, protein stability, proteasome, polyubiquitin receptor

## Abstract

The dynamic balance of transcriptional and translational regulation together with degron-controlled proteolysis shapes the ever-changing cellular proteome. While a large variety of degradation signals has been characterized, our knowledge of *cis*-acting protein motifs that can in vivo stabilize otherwise short-lived proteins is very limited. We have identified and characterized a conserved 13-mer protein segment derived from the p54/Rpn10 ubiquitin receptor subunit of the *Drosophila* 26S proteasome, which fulfills all the characteristics of a protein stabilization motif (STABILON). Attachment of STABILON to various intracellular as well as medically relevant secreted model proteins resulted in a significant increase in their cellular or extracellular concentration in mammalian cells. We demonstrate that STABILON acts as a universal and dual function motif that, on the one hand, increases the concentration of the corresponding mRNAs and, on the other hand, prevents the degradation of short-lived fusion proteins. Therefore, STABILON may lead to a breakthrough in biomedical recombinant protein production.

## 1. Introduction

Controlled intracellular proteolysis by the ubiquitin–proteasome system (UPS) and autophagy are essential for the maintenance of the healthy proteome (proteostasis) in Eukaryotes. UPS is responsible for the elimination of short-lived proteins, including transcription factors or cell cycle regulators, which are frequently involved in critical regulatory functions; therefore, their selective and orchestrated destruction is essential for cellular integrity. Degradation signals (Degrons) are present in short-lived proteins ensuring the selective recognition, polyubiquitination and elimination of these polypeptides by the UPS. Degrons are either short intramolecular segments of proteins or short amino acid sequences at the N-terminus (N-end rule degrons) or C-terminus (C-end degrons) of the target protein that are recognized by E3 ubiquitin ligases under different conditions [1,2,3].

Polyubiquitinated substrates of the UPS are recognized by the receptor subunits of the 26S proteasome (Rpn10, Rpn13 and Rpn1) in cooperation with extraproteasomal ubiquitin receptor proteins (Rad23, Dsk2 and Ddi1) [4,5,6,7]. Previously, we have identified, cloned and characterized p54 (UniProt ID: P55035; orthologue of the yeast Rpn10 and human S5a/PSMD4), the main polyubiquitin receptor subunit of the *Drosophila* 26S proteasome [8,9]. Deletion of the single copy *p54* gene in *Drosophila* (termed as *Δp54*) caused dramatic phenotypic changes and polyphasic lethality, which could be fully rescued by the expression of a wild-type *p54* in the null mutant background [8]. We observed that the extreme C-terminus (aa. 382-KDKDKKSDGKDSQKK-396) of the p54 protein that is composed of seven lysine residues (named as K-rich cluster) interspersed with acidic amino acids is highly conserved [5]. The deletion of this sequence from full-length p54 (p54-ΔK) did not affect the substrate-binding ability and proteasomal localization of p54-ΔK but only partially rescued the *Δp54*-caused lethality, suggesting that this motif is essential [5]. Moreover, in transgenic *Drosophila* lines overexpressing only the C-terminal half of subunit p54 (CTH) or its derivative from which the last 15 amino acids had been deleted (CTH-ΔK), we noticed that while the cellular concentration of the CTH was comparable to the endogenous p54 level, the concentration of CTH-ΔK was orders of magnitude lower [4,5]. This suggested that the K-rich cluster might serve as a stabilization sequence; therefore, to the analogy of degrons, we named it STABILON (abbr. Stab).

While a large variety of degradation signals have been discovered, our knowledge of *cis*-acting protein motifs that can in vivo stabilize otherwise short-lived or recombinant proteins expressed in heterologous systems is very limited. The discovery of stabilization motifs with antagonistic effects on degrons would open new research avenue in the field of proteostasis, moreover, would revolutionize the production of therapeutic proteins (e.g., monoclonal antibodies, hormones, etc.) in insect and mammalian tissue cultures. Therefore, we further investigated the conserved and essential STABILON sequence of p54.

In this work, we demonstrate that the extreme difference in the concentrations of the transgenic CTH and CTH-ΔK (hereafter CTH-ΔStab) proteins is generated by two independent regulatory events: (i) the STABILON motif provides an increased mRNA concentration of the transgenic CTH construct compared with that of the CTH-ΔStab; (ii) STABILON can significantly extend the in vivo half-life of the CTH protein by retarding its degradation. Furthermore, we prove that this motif is a universal and dual function *cis*-acting signal, and its attachment to several intracellular or biotechnologically relevant secreted proteins significantly elevates the amount of the fusion proteins in *Drosophila* as well as in CHO mammalian cell cultures. Our results demonstrate that STABILON would be a superior tool in heterologous protein production in insect or mammalian tissue cultures.

## 2. Results

### 2.1. The Proteasome Subunit p54/Rpn10 Harbors a Conserved C-Terminal Stabilization Motif

**Figure 1:**
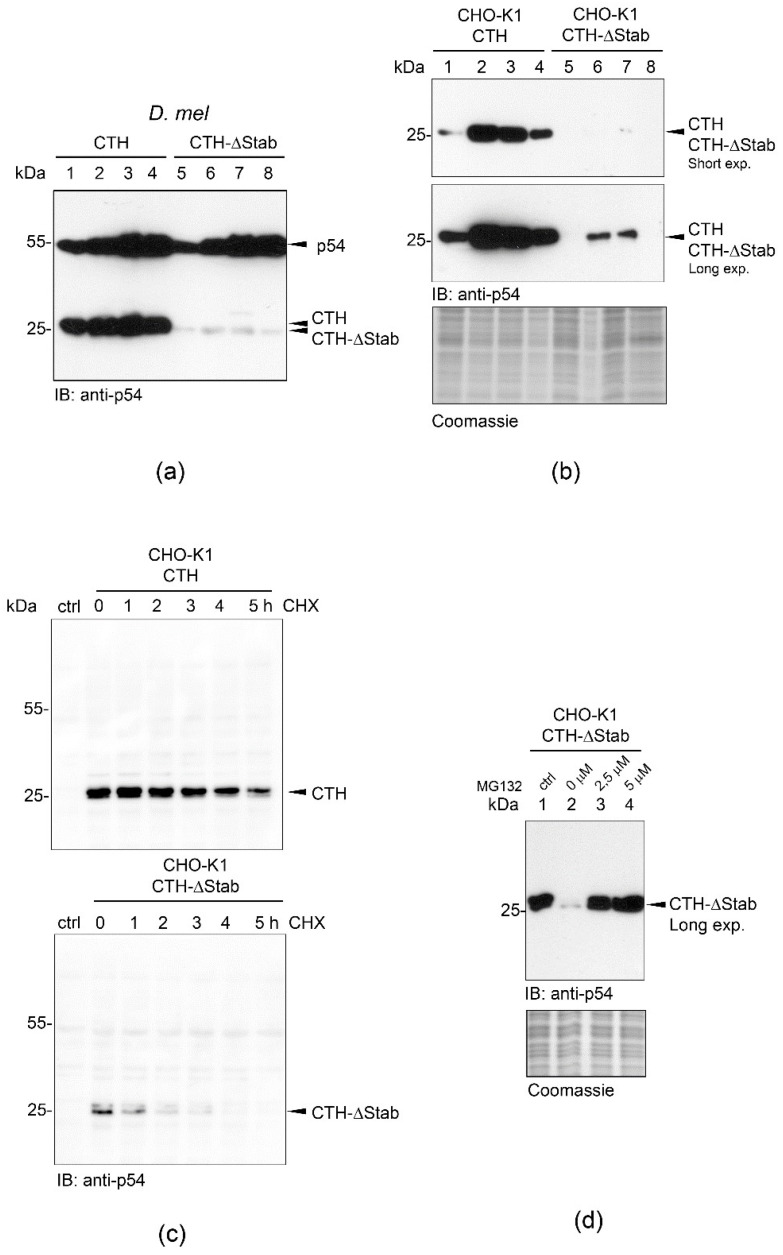
High-level accumulation and intracellular stability of CTH are STABILON-dependent. (**a**) *CTH* (lanes 1–4) or *CTH-∆Stab* (lanes 5–8) transgene expression was analyzed in total protein extracts prepared from 2nd (lane 1 and 5) or 3rd instar larvae (lanes 2 and 6), pupae (lane 3 and 7) or adults (lane 4 and 8), respectively, by immunoblotting using CTH-specific monoclonal antibody (anti-p54). Endogenous p54 serves as loading control. (**b**) Stably transfected CHO-K1 cells expressing CTH (lanes 1–4) or CTH-∆Stab (lanes 5–8) proteins were grown without doxycycline (non-induced, lanes 1 and 5) or in the presence of doxycycline for 24 h (induced, lanes 2 and 6) or 24 h post-induction further incubated in doxycycline-free media for an additional 24 h (lanes 3 and 7) and 48 h (lanes 4 and 8), respectively. Total protein extracts were analyzed by immunoblotting (short and long exposure are shown). Coomassie-stained gel represents the protein loading. (**c**) Cycloheximide chase experiment showing that STABILON is needed for the extended half-life of CTH. Stably transfected CHO-K1 cells expressing *CTH* or *CTH-ΔStab* transgenes were doxycycline-induced for 20 h, and cells were further grown in doxycycline-free medium for 0–5 h in the presence of cycloheximide (CHX). Cells were harvested and subjected to immunoblotting. Non-induced cells serve as control (ctrl). (**d**) CTH-ΔStab protein expression was induced with doxycycline in stably transfected CHO-K1 cells for 16 h (lane 1). In lanes 2–4, doxycycline-induced cells were further incubated for 10 h in doxycycline-free medium in the presence of 0 µM (lane 2), 2.5 µM (lane 3) or 5 µM (lane 4) MG132 proteasome inhibitor, respectively. Total protein extracts were subjected to SDS-PAGE followed by immunoblotting (long exposure is shown). Coomassie-stained gel shows protein loading.

We have shown previously that the C-terminal 15-mer tail of the proteasome subunit p54/Rpn10 is conserved from flies to man (Figure S1), and its presence is essential for the function of the proteasome [5]. In order to dissect the exact role of this segment, we have generated in this study a large number of p54-CTH- or p54-CTH-∆Stab (lacking the last 15 amino acids)-expressing UAS-Gal4-regulated [10] transgenic *Drosophila melanogaster* lines. For the unambiguous and comparable analysis of the transcriptional or posttranscriptional regulation of transgenic protein profiles, transgenes were inserted into the same, well-characterized genomic locus by landing-site targeted integration [11]. Surprisingly, while a high level of the CTH protein was seen in all lines, the CTH-ΔStab was barely detectable (in any of the twenty-four independent lines) by Western blotting using a highly specific monoclonal antibody recognizing a short epitope present in both CTH and CTH-ΔStab proteins (hereafter anti-p54 mAb [4]). Moreover, the concentration of the transgenic CTH was high in all tested ontogenetic stages, comparable to that of the endogenous full-length p54 protein, while CTH-∆Stab protein was almost undetectable in any developmental stages (Figure 1a). This result suggested that the C-terminal tail of p54/Rpn10 is needed for the integrity (or stability) of the protein; therefore, we named it STABILON.

To approach the molecular mechanism leading to these differences, we monitored the in vivo half-life of the CTH and CTH-ΔStab proteins in mammalian cells. We chose the tetracycline/doxycycline-inducible CHO-K1 Tet-On cell system, which allows rapid transcriptional induction of the target genes by the addition of doxycycline (inducer), and sudden shutdown of transcription by the removal of the inducer from the culture medium. In addition, this system is suitable for the in vivo testing of the effect of the fruit fly STABILON sequence in higher Eukaryotes. Therefore, we established *Drosophila* CTH or CTH-ΔStab-expressing stably transfected CHO-K1 cells by standard procedures. The transfection efficiency was very high for both constructs generating several thousand independent stable transfected cells, which were pooled to minimalize the probability of insertion site-dependent transcriptional differences of transgene expression. Transgene expression was induced for 24 h, cells were further grown for 0, 24 and 48 h in a doxycycline-free medium (transcriptional shutdown), and the cellular concentration of CTH or CTH-∆Stab proteins were analyzed by Western blotting (Figure 1b). We found that the amount of CTH, just like in the *Drosophila* transgenic model (Figure 1a), was orders of magnitude higher in these mammalian cells than that of the CTH-ΔStab protein (Figure 1b). Moreover, transcriptional shutdown revealed that a large proportion of the CTH protein was preserved even 48 h post-induction, while the CTH-ΔStab protein was completely eliminated during this period (compare lanes 4 and 8 in Figure 1b). This suggests that STABILON is needed for the stabilization and extended half-life of CTH.

### 2.2. STABILON Is Required for the Extended Half-Life of p54-CTH

In order to precisely analyze the STABILON-dependent in vivo half-life of CTH, we performed a cycloheximide chase experiment in CHO-K1 cells. CTH or CTH-ΔStab-expression was induced for 20 h, and then cell cultures were further grown in the doxycycline-free medium in the presence of the translation inhibitor cycloheximide for 0, 1, 2, 3, 4 and 5 h. Cells were harvested, and the cellular amount of CTH or CTH-ΔStab was analyzed by Western blotting. This experiment has clearly demonstrated that the huge difference in the protein levels of CTH and CTH-∆Stab is a consequence of a remarkably shorter half-life of the CTH-ΔStab protein (Figure 1c). Band intensity measurements have revealed that while 59% of the STABILON-containing CTH was still detectable 5 h post-cycloheximide-treatment, the CTH-ΔStab protein had completely disappeared after 4 h, and its half-life (50%) was only around 1 h (Figure S2).

Strikingly, we found that the shorter half-life of the CTH-ΔStab protein is dependent on the activity of the proteasome. Treatment of the stable cell lines with MG132, a selective inhibitor of the 20S proteasome, has slowed down the rapid decline of the CTH-∆Stab protein (Figure 1d). We have shown previously that the C-terminal half of p54 is disordered [12], and it is known that many unstructured proteins (or protein segments) can be eliminated in a ubiquitin-independent manner by the 20S catalytic complex of the proteasome [13]. This also suggests that the STABILON tail is needed for the protection of CTH while feeding the ubiquitinated substrates into the catalytic chamber of the 20S proteasome.

### 2.3. STABILON Is Not a Weak Initiation Sequence but a Genuine Cis-Acting Stabilization Motif

**Figure 2:**
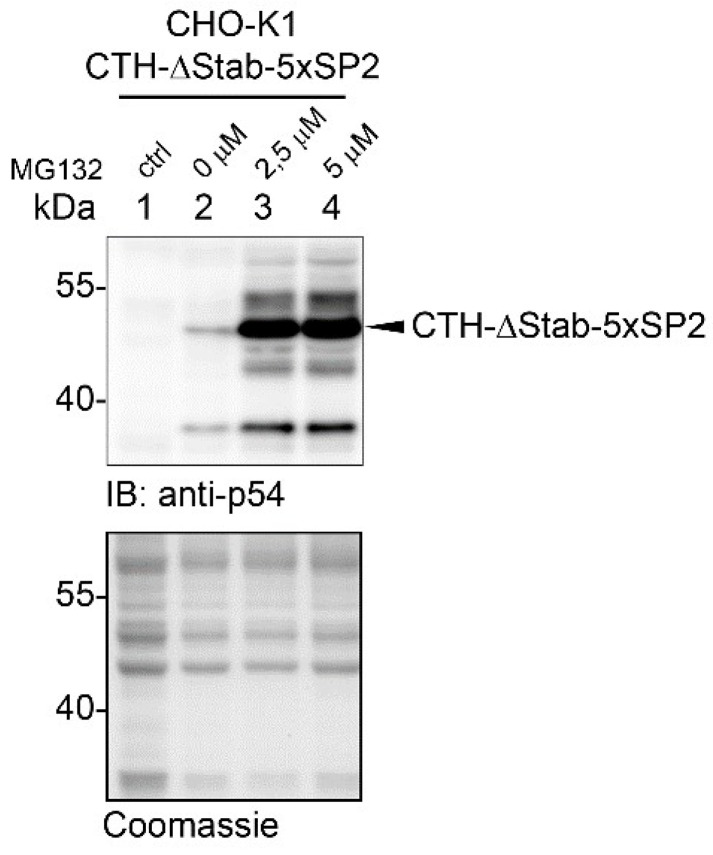
The weak initiator sequence 5 × SP2 does not have any effect on the accumulation of intracellular CTH-ΔStab in CHO-K1 cells. Accumulation of CTH-ΔStab-5 × SP2 fusion protein in non-induced (lane 1) or doxycycline-induced (for 24 h) stably transfected CHO-K1 cells treated with 0 µM (lane 2), 2.5 µM (lane 3) or 5 µM MG132 (lane 4), respectively. Protein extracts were analyzed by immunoblotting. Coomassie-stained gel shows protein loading.

It has recently been shown that besides polyubiquitination, proteasomal degradation of proteins is also regulated by initiation sequences located at the C-terminus of the target protein [13]. The proteasome engages its substrates at initiator sites, which is a prerequisite step for the degradation of the target. Depending on the amino acid composition of the initiators, they may slow down (weak initiators) or boost (strong initiators) the rate of proteasomal degradation [13]. We aimed to prove that STABILON is not a weak initiator sequence but utilizes a different mechanism to stabilize proteins. To this end, we fused five copies of the SP2 motif (a segment of Influenza A M2 protein [13]), the weakest initiator sequence so far characterized, to the C-terminal end of CTH-ΔStab protein. The accumulation of the fusion protein was analyzed in stably transfected CHO-K1 cells in the absence or presence of MG132 proteasome inhibitor (Figure 2). We hypothesized that if STABILON acts as a weak initiator, its replacement in the CTH protein with the 5 × SP2 motif will increase the concentration of CTH-ΔStab-5 × SP2 to the level of CTH. However, we observed the opposite. In the absence of MG132, only trace amounts of CTH-ΔStab-5 × SP2 protein were detected by immunoblotting (Figure 2), similarly to the levels of CTH-ΔStab (Figure 1d). The huge accumulation of the CTH-ΔStab-5 × SP2 fusion protein in the presence of MG132 clearly indicates the role of the proteasome in the elimination of CTH-ΔStab-5 × SP2 (Figure 2). All these results suggest that STABILON is not a weak initiator.

### 2.4. STABILON Prevents the Proteasomal Degradation of Short-Lived Proteins

**Figure 3:**
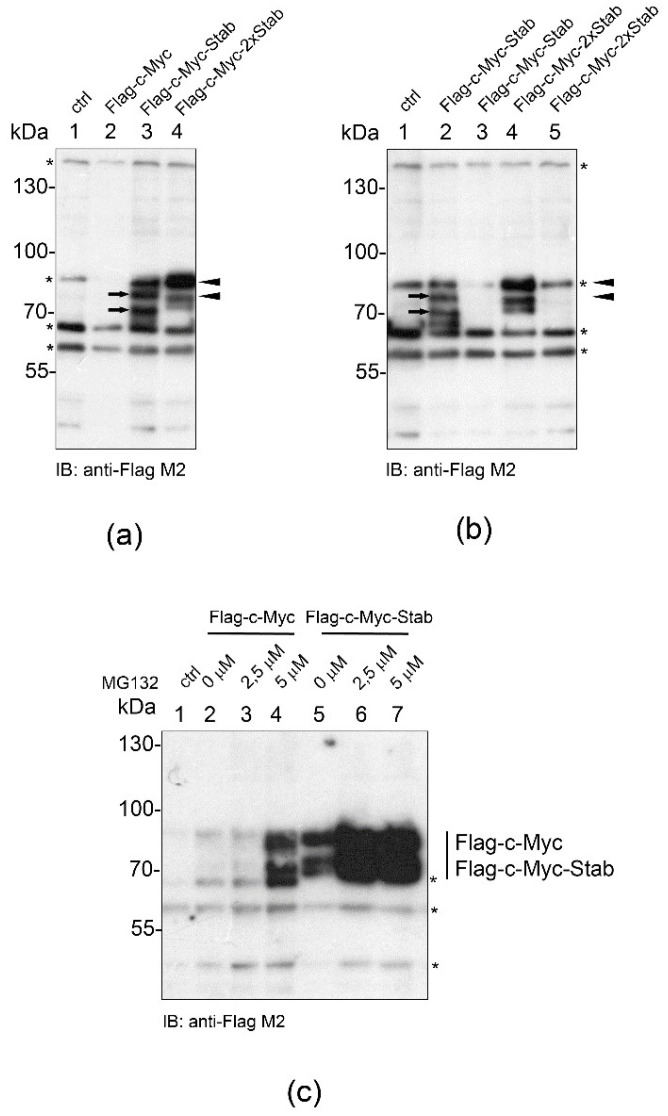
STABILON transiently stabilizes the transcription factor c-Myc. (**a**) Stably transfected CHO-K1 cells expressing Flag-c-Myc (lane 2), Flag-c-Myc-Stab (lane 3) or Flag-c-Myc-2 × Stab proteins (lane 4), respectively, were induced by doxycycline treatment for 24 h and analyzed by immunoblotting. Total protein extract of non-transformed CHO-K1 cells serves as negative control (lane 1). Arrows indicate Flag-c-Myc-Stab, while arrowheads mark the Flag-c-Myc-2 × Stab proteins. Non-specific bands (marked by asterisks) serve as loading control. (**b**) Stably transfected CHO-K1 cells expressing Flag-c-Myc-Stab (lanes 2 and 3) or Flag-c-Myc-2 × Stab proteins (lanes 4 and 5) were induced by doxycycline treatment for 24 h. Flag-c-Myc-Stab (lane 3) and Flag-c-Myc-2 × Stab (lane 5) cells were further incubated in doxycycline-free medium for 24 h before subjected to immunoblotting using anti-FlagM_2_ monoclonal antibody. Arrows mark Flag-c-Myc-Stab, while arrow heads indicate the Flag-c-Myc-2 × Stab proteins. Non-specific bands (marked by asterisks) serve as loading control. Protein extract of non-transformed CHO-K1 cells serves as negative control (lane 1). (**c**) Stably transfected CHO-K1 cells expressing the Flag-c-Myc (lanes 2–4) or Flag-c-Myc-Stab (lanes 5–7) proteins were grown in the presence of doxycycline for 10 h, and 4 h post induction cell culture media were supplemented with 0 µM (lanes 2 and 5), 2.5 µM (lanes 3 and 6) or 5 µM (lanes 4 and 7) of MG132 proteasome inhibitor. Total protein extracts were fractionated on SDS-PAGE and analyzed by immunoblotting. Protein extract of CHO-K1 cells is shown as control (lane 1), while non-specific bands (marked by asterisks) serve as loading control.

We aimed to analyze whether STABILON confers the stabilization effect on other well-characterized short-lived proteins, too. We chose c-Myc, a transcription factor controlling several essential cellular processes [14] because it has an extended intrinsically unstructured region and its concentration is tightly regulated by a variety of post-translational modifications, which influence its proteasome-dependent degradation [15,16]. In order to counteract the very short half-life of c-Myc, two derivatives of the N-terminally Flag-tagged c-Myc construct were created in which a single copy (-Stab) or two copies (-2 × Stab) of the STABILON sequence were fused to the C-terminus of the transgenes. Stably transfected CHO-K1 cells were selected, the protein expression was induced, and accumulation of Flag-c-Myc, Flag-c-Myc-Stab or Flag-c-Myc-2 × Stab proteins was analyzed by immunoblotting. Only trace amounts of Flag-c-Myc were detected 24 h post-induction. In contrast, Flag-c-Myc-Stab and Flag-c-Myc-2 × Stab were accumulated at high levels, respectively (Figure 3a). Although the presence of STABILON was required for the accumulation of Flag-c-Myc-Stab and Flag-c-Myc-2 × Stab, they remained relatively short-lived because removal of doxycycline from the culture medium 24 h post-induction resulted in complete elimination of the synthesized proteins during a second, induction-free 24 h-long period (Figure 3b). This elimination is unequivocally a proteasome-dependent proteolytic process because increasing concentrations of MG132 boosted the accumulation of Flag-c-Myc and Flag-c-Myc-Stab (Figure 3c), respectively. This result further supports the observation that STABILON protects the fusion proteins from proteasomal degradation and boosts their intracellular accumulation in mammalian cells.

### 2.5. STABILON Enhances the Accumulation of Globular and Disordered Proteins

**Figure 4:**
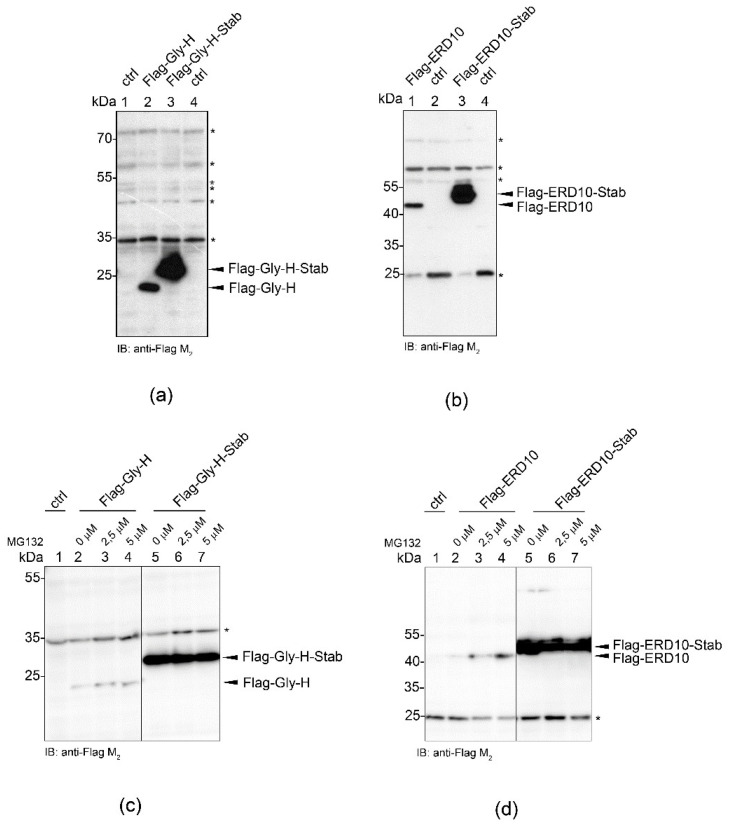
STABILON increases the cellular concentration of globular and intrinsically unstructured proteins in mammalian cells. (**a**) Expression of Flag-tagged Gly-H or Gly-H-Stab proteins was induced in CHO-K1 cells by doxycycline treatment for 24 h. Total protein extracts of control CHO-K1 cells (lanes 1 and 4; ctrl), Flag-Gly-H (lane 2) or Flag-Gly-H-Stab (lane 3) cells, respectively, were fractionated on SDS-PAGE and analyzed by immunoblotting. Non-specific bands (marked with asterisks) serve as loading control. (**b**) Expression of Flag-tagged intrinsically unstructured ERD10 or ERD10-Stab proteins was induced in CHO-K1 cells by doxycycline treatment for 24 h. Total protein extracts of control CHO-K1 cells (lanes 2 and 4; ctrl), ERD10 (lane 1) or ERD10-Stab cells (lane 3), respectively, were fractionated on SDS-PAGE and analyzed by immunoblotting. Non-specific bands (marked by asterisks) serve as loading control. (**c**) Expression of the globular Flag-Gly-H (lanes 2–4) or Flag-Gly-H-Stab (lanes 5–7) proteins was induced in stably transfected CHO-K1 cells by 24 h doxycycline treatment. During the last 10 h of induction, the medium was supplemented with 0 μM (lanes 2 and 5), 2.5 μM (lanes 3 and 6) or 5 μM (lanes 4 and 7) MG132 proteasome inhibitor. CHO-K1 cell extract serves as control (lane 1). Total cell extracts were fractionated on SDS-PAGE and analyzed by immunoblotting. Non-specific band (marked by asterisk) serves as loading control. (**d**) Expression of the intrinsically unstructured Flag-ERD10 (lanes 2–4) or Flag-ERD10-Stab (lanes 5–7) proteins were induced in stably transfected CHO-K1 cells by 24 h doxycycline treatment. During the last 10 h of induction, the medium was supplemented with 0 μM (lanes 2 and 5), 2.5 μM (lanes 3 and 6) or 5 μM (lanes 4 and 7) MG132 proteasome inhibitor. CHO-K1 cell extract serves as control (lane 1). Total cell extracts were fractionated on SDS-PAGE and analysed by immunoblotting. Non-specific band (marked by asterisk) serve as loading control.

To better understand the mode of action of the STABILON motif, we investigated whether it can protect intrinsically unstructured proteins exclusively (such as CTH or c-Myc) or it can also influence the fate of globular proteins. A pair of well-characterized model proteins was chosen to test the in vivo effect of the STABILON: the intrinsically unstructured ERD10 from *Arabidopsis thaliana* (Dehydrin ERD10, UniProt P42759) and the globular Gly-H from *Escherichia coli* (Glycine cleavage system H protein, UniProt P0A6T9) [12,17]. Neither are encoded in the fly or CHO genomes. We found that the attachment of the STABILON tag resulted in a huge increase in the concentration of the ordered Gly-H (Figure 4a) as well as the disordered ERD10 proteins (Figure 4b) compared to their untagged forms in mammalian cells. However, surprisingly, we also showed that this was independent of the proteasome because MG132-treatment did not change the cellular concentrations of any of the four tested proteins (Figure 4c,d). We, therefore, suppose that some proteins, albeit not genuine substrates of the proteasomal degradation system, are still influenced by the STABILON motif at the protein level. These observations indicate that an intrinsically unstructured character of a protein alone does not explain the mode(s) of action of the STABILON motif.

### 2.6. STABILON Boosts the Expression of Secreted Proteins

**Figure 5:**
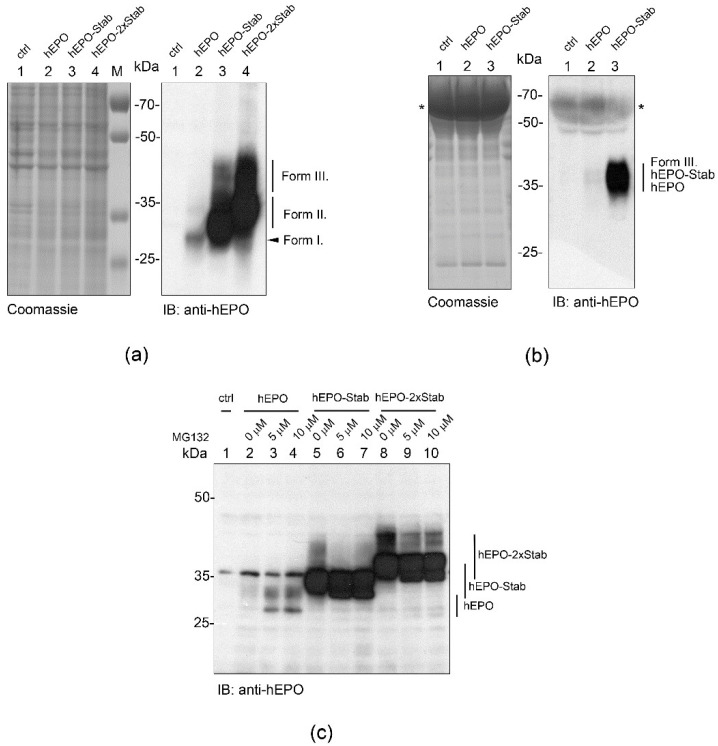
STABILON significantly boosts the intracellular and secreted levels of hEPO in mammalian cells. (**a**) Stably transfected CHO-K1 cells expressing hEPO (lane 2), hEPO-Stab (lane 3) or hEPO-2 × Stab (lane 4) proteins were induced by doxycycline treatment for 24 h. Lane 1 is the control nontransfected cell. Total cell extracts were fractionated on SDS-PAGE. Expression of hEPO derivatives (unmodified and glycosylated forms, Form I–III) was analyzed by immunoblotting using anti-hEPO monoclonal antibody. Coomassie-stained gel shows the protein loading (left panel). (**b**) 10 µL of cell culture medium of doxycycline-induced control (lane 1), hEPO (lane 2) or hEPO-Stab (lane 3) expressing stably transfected CHO-K1 cells were fractionated on SDS-PAGE. Secreted hEPO glycosylated species (Form III) were analyzed by immunoblotting. Coomassie-stained gel serves as loading control (left panel). Asterisk labels the extremely abundant serum albumin present in the medium. (**c**) Stable transfected CHO-K1 cells expressing hEPO, hEPO-Stab or hEPO-2 × Stab were induced with doxycycline treatment for 5 h, then continued for an additional 5 h in the presence of 0 μM (lanes 2, 5 and 8), 5 μM (lanes 3, 6 and 9) and 10 μM (lanes 4, 7 and 10) of MG132. Accumulation of hEPO derivatives was analyzed in total cell extracts by immunoblotting. Protein extracts of CHO-K1 cells served as control (lane 1).

Besides cytosolic proteins, we have tested the *cis*-acting effect of STABILON on the expression of human erythropoietin (hEPO), a secreted and glycosylated hormone protein. Several interesting properties of hEPO justify our choice: (i) it is a medically relevant secreted protein that needs to be expressed in mammalian cells for proper glycosylation essential for its physiological activity, and (ii) it contains ordered and intrinsically unstructured segments [18]. The human EPO or its derivatives with one or two copies of STABILON fused at their C-terminus were cloned into the pTRE2hyg vector to generate stably transfected CHO-K1 cells under the control of the Tet-On system. After doxycycline induction, the cellular level and the processing of the recombinant proteins were analyzed in total CHO-K1 cell lysates, while fully processed, glycosylated and secreted hEPO and its derivatives were detected in the tissue culture medium by immunoblotting using an anti-hEPO monoclonal antibody. As expected, the intracellular concentration of the hEPO-Stab and hEPO-2 × Stab was orders of magnitude higher than that of the nascent hEPO protein (Figure 5a). The insertion of a second STABILON motif only slightly boosted the yield of the recombinant protein compared to the single-tagged hEPO-Stab. More importantly, the effect of STABILON was more dramatic comparing the quantities of the hEPO and hEPO-Stab proteins secreted into the tissue culture medium (Figure 5b).

Immunoblotting has revealed that three populations of hEPO or its derivatives were present in the total cell lysates (Figure 5a). Form I and II are the non-glycosylated and the primarily glycosylated forms of hEPO modified in the endoplasmic reticulum, respectively [18]. Form III is the fully glycosylated hEPO protein that is postsynthetically modified in the endoplasmic reticulum and the Golgi complex prior to secretion. The electrophoretic mobility of Form III is indistinguishable from that of the secreted (matured) protein form (Figure 5b), and its molecular mass is in accordance with the literature [18]. Moreover, mass spectrometric analysis of the secreted hEPO-Stab protein (with Form III glycosylation) in the culture medium has validated that the protein lacks the N-terminal signal peptide, hence normally went through the secretory pathway (Figure S3). Despite the low yield of secreted hEPO its electrophoretic mobility still suggests that it is properly glycosylated (Form III). All these suggest that the C-terminal STABILON tail significantly boosts the level of secreted hEPO without disturbing its post-translational modification and maturation.

Treatment of cells with increasing concentrations of MG132 yielded more intracellular nascent hEPO, while MG132 had only a minor effect on the intracellular concentration of hEPO-Stab or hEPO-2 × Stab proteins (Figure 5c).

### 2.7. Mapping the Minimal STABILON

**Figure 6:**
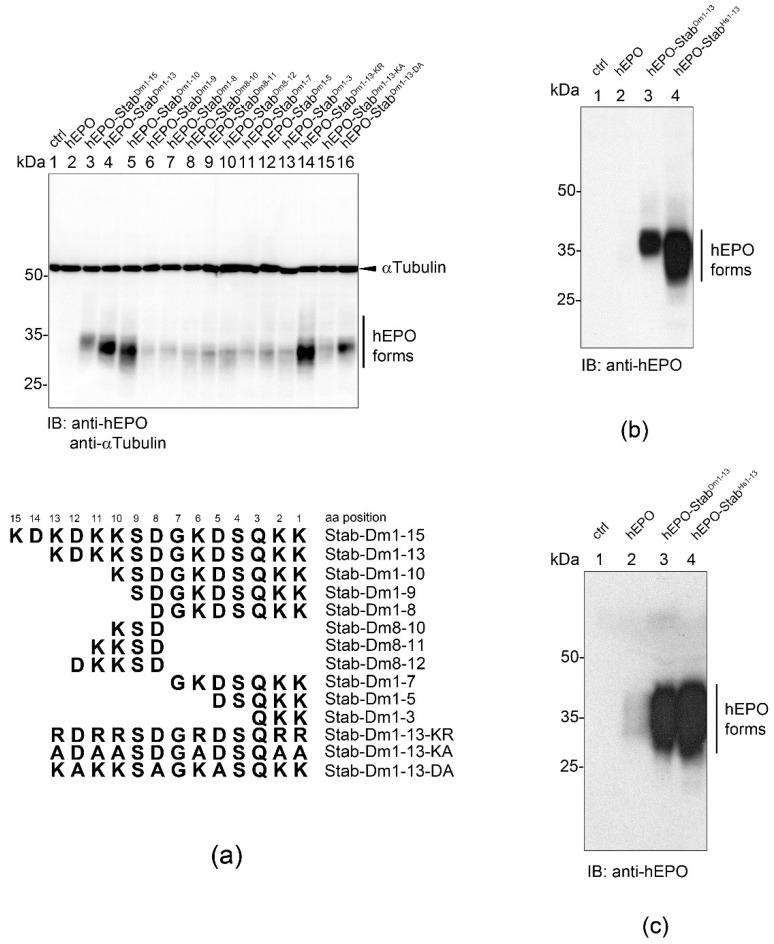
The effect of STABILON derivatives on the accumulation of hEPO in mammalian cells. (**a**) hEPO (lane 2), hEPO-Stab^Dm1−15^ (lane 3), hEPO-Stab^Dm1−13^ (lane 4), hEPO-Stab^Dm1−10^ (lane 5), hEPO-Stab^Dm1−9^ (lane 6), hEPO-Stab^Dm1−8^ (lane 7), hEPO-Stab^Dm8−10^ (lane 8), hEPO-Stab^Dm8−11^ (lane 9), hEPO-Stab^Dm8−12^ (lane 10), hEPO-Stab^Dm1−7^ (lane 11), hEPO-Stab^Dm1−5^ (lane 12), hEPO-Stab^Dm1−3^ (lane 13), hEPO-Stab^Dm1−13-KR^ (lane 14), hEPO-Stab^Dm1−13-KA^ (lane 15) and hEPO-Stab^Dm1−13-DA^ (lane 16) transgenes in stably transfected CHO-K1 cells were induced by doxycycline-treatment for 24 h, respectively. The intracellular accumulation of hEPO derivatives was analyzed in total cell extracts by Western blotting using anti-hEPO monoclonal antibody. αTubulin served as loading control. The lower diagram shows the amino acid positions and composition of the STABILON derivatives fused to hEPO. (**b**) hEPO (lane 2), hEPO-Stab^Dm1-13^ (lane 3) or hEPO-Stab^Hs1–13^ (lane 4) were expressed in stably transfected CHO-K1 cells by doxycycline-treatment for 24 h. Accumulation of hEPO derivatives was analyzed in total cell extracts by Western blotting using anti-hEPO monoclonal antibody. Protein extracts of CHO-K1 cells serve as control (lane 1). (**c**) Stably transfected CHO-K1 cells carrying the hEPO (lane 2), hEPO-Stab^Dm1−13^ (lane 3) and hEPO-Stab^Hs1−13^ (lane 4) genes were induced by doxycycline for 24 h. hEPO derivatives secreted into the tissue culture medium were analyzed by Western blotting with anti-hEPO monoclonal antibody. Tissue culture medium of CHO-K1 cells served as control (lane 1).

To delineate the essential sequence(s) required for elevated hEPO production, we fused sequentially shortened versions of the STABILON to the C-terminus of hEPO and analyzed the accumulation of the intracellular hEPO derivatives expressed in stably transfected CHO-K1 cells. Removal of the N-terminal first two amino acids (KD) of the 15-mer STABILON improved the accumulation of the hEPO-Stab^Dm1-13^ protein (Figure 6a). With this screen, we found that the last 10 amino acids of STABILON (hEPO-Stab^Dm1-10^) are absolutely essential for the high-level accumulation of hEPO-derivatives, while shorter sequences drastically destroyed the potency of STABILON derivatives (Figure 6a, lanes 1–13).

Multiple sequence alignments of the very C-terminal sequences of the proteasomal polyubiquitin receptor subunits (p54/Rpn10 orthologues available at Ensemble release 106) in primates, other mammals or *Drosophilidae* (Figure S1) revealed that the lysine and acidic amino acid residues are highly conserved. Therefore, we replaced all lysines in STABILON^Dm1−13^ with arginine (STABILON^Dm1−13-KR^), which is physicochemically similar to lysines, and found that this modification only slightly impaired the ability of the motif to boost the in vivo accumulation of hEPO (Figure 6a, compare lane 14 to lane 4). In contrast, the replacement of all lysine residues with uncharged alanines (STABILONDm^1−13-KA^) almost completely destroyed the potency of STABILONDm1-13 (Figure 6a, compare lane 15 to lane 4). Finally, we demonstrated that the conserved acidic amino acid residues are also essential because their mutation to alanines (hEPO-Stab^Dm1−13-DA^) suppressed the accumulation of hEPO in CHO-K1 cells (Figure 6a, compare lane 16 to lane 4).

The 13-mer STABILON present in the p54 polyubiquitin receptor subunit of the 26S proteasome is highly conserved from *Drosophila* to humans (Figure S1) [5]. Therefore, we aimed to test the C-terminal 13 amino acid-long sequence (365-KDGKKDKKEEDKK-377, UniProt ID: P55036) of the human S5a/PSMD4 proteasomal subunit for its ability to boost hEPO production when fused to its C-terminus. As expected, the quantity of the intracellular and secreted hEPO-Stab^Hs1−13^ proteins was orders of magnitude higher than that of the nascent hEPO (Figure 6b,c). Consequently, the 13-amino acid long STABILON is an evolutionarily conserved, genuine, *cis*-acting and universal protein stabilization motif.

### 2.8. STABILON Affects the Transcript Levels of the Fusion Proteins

To better understand the mechanism that boosts the amount of the STABILON-fused model proteins, we analyzed the transcript levels of the various transgenes by quantitative RT-PCR in stably transfected non-induced or doxycycline-induced cell lines, respectively. We found that in all cases, the mRNA concentrations of the STABILON-fused transgenes were 4–15-times higher than that of the parental transgenes (Figure S4, doxycycline-induced). Moreover, we found that in stably transfected cells, the mRNA concentrations of the STABILON-fused transgenes are 2.5–5.5-times higher even in the absence of an inducer (Figure S4, non-induced).

In the widely used Tet-On system, transgene expression is blocked in the absence of a doxycycline inducer. Upon induction, the CHO-K1 genome-encoded Tet repressor dissociates from the *tet* operator DNA elements present in the promoter region, which then drives transgene expression. The expression of the STABILON-fused transgenes in non-induced cells may indicate that the presence of the DNA sequence encoding the STABILON motif overrides the Tet-repressor generated transcriptional blockade, i.e., it functions as a transcriptional enhancer. The analysis of a different set of transgene constructs definitely precludes this assumption. In all the above-described transgene constructs, an in-frame stop codon (hereafter STOP) at the 3′-end of the coding sequence abutted by the DNA sequence encoding the STABILON motif completely eliminated the STABILON-induced protein overproduction. This means that the STABILON motif must be present within the translated region of the mRNA.

**Figure 7:**
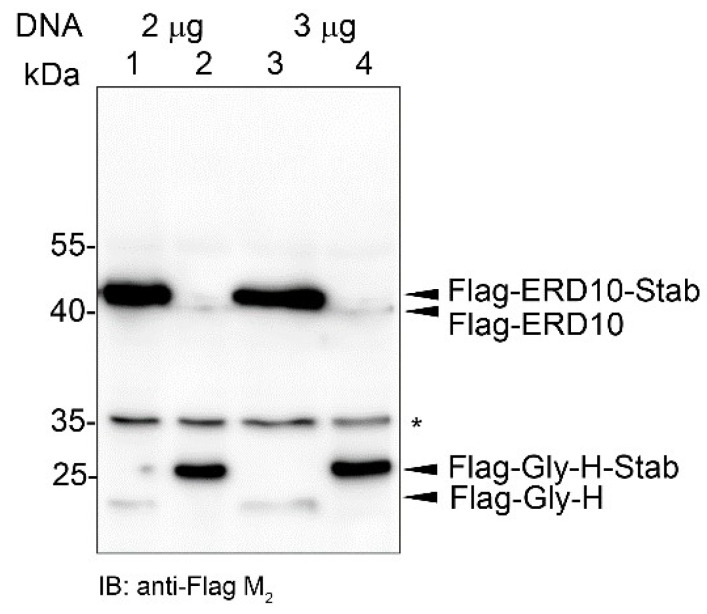
STABILON fusion is the prerequisite for efficient transgene expression in non-induced transiently transfected CHO-K1 cells. CHO-K1 cells transiently transfected with a mixture of Flag-Gly-H and Flag-ERD10-Stab (lane 1 (2 μg DNA from each), lane 3 (3 μg DNA from each)) or Flag-Gly-H-Stab and Flag-ERD10 (lane 2 (2 μg DNA from each), lane 4 (3 μg DNA from each)), respectively, were analyzed by immunoblotting 20 h post-transfection. Non-specific band (marked by asterisk) serves as loading control.

To further corroborate the significance of the STABILON-generated regulation, CHO-K1 cells were transiently transfected with a mixture of equal amounts of *Flag-Gly-H-STOP-Stab* and Flag-ERD10-Stab-STOP, or Flag-Gly-H-Stab-STOP and *Flag-ERD10-STOP-Stab DNAs*, respectively. Twenty hours post-transfection (without induction with doxycycline), cells were collected, and the accumulation of Flag-Gly-H/Flag-ERD10-Stab or Flag-Gly-H-Stab/Flag-ERD10 proteins was analyzed by immunoblotting. We found that in both transient transfection experiments, only those members of the co-transfected genes generated high-level protein production in which the STABILON motif was present in the translated region of the mRNA (Figure 7).

It is reasonable to suppose that the presence of the STABILON sequence in the translated region of the mRNA may slow down the 3′–5′ decay of the mRNA and thus boost the accumulation of the mRNAs, which appear as a consequence of a minimally leaky Tet-repressor-controlled promoter [19]. To prove this assumption, we analyzed the rate of mRNA decay in hEPO-STOP-Stab- or hEPO-Stab-STOP-expressing stably transfected CHO-K1 cells. Following 20 h induction with doxycycline the cells were grown for 2 h in a doxycycline-free medium to allow to decline the transcription of the transgenes and further incubated in doxycycline-free medium for 0, 4, 8 or 10 h. At the indicated time points, the level of the corresponding mRNAs was measured by quantitative RT-PCR (Figure S5). The 10 h half-life of the *hEPO-Stab-STOP* mRNA corresponds to the half-life of the most stable mRNAs (e.g., *globin* mRNA [20]). The exact measurement of the half-life of the *hEPO-STOP-Stab* mRNA, which is expressed at a very low level, may be seriously disturbed by its low level but continuous expression due to the minimally leaky Tet-repressor-controlled promoter. It will be a future challenge to investigate the molecular details of mRNA decay kinetics regulated by the STABILON motif.

## 3. Discussion

Previously, we have constructed a UAS-Gal4-regulated transgenic *Drosophila* line overexpressing the C-terminal half of subunit p54 (CTH) [4,5]. As expected, due to the lack of the N-terminal vWA domain of p54 that is responsible for the proteasomal incorporation of the subunit, transgenic CTH was not assembled into the proteasome but accumulated extraproteasomally. We have shown that the lysine-rich (K-rich) cluster present at the C-terminal end of CTH is essential and involved in the extraproteasomal function of the subunit [4,5]. In the present study, we demonstrate that the C-terminal K-rich sequence has another important function. It also serves as a *cis*-acting protein stabilization signal; therefore, we named it STABILON. We also found that intrinsically unstructured and globular proteins fused to either the *Drosophila* or the very similar human STABILON are protected against degradation, prolonging the in vivo half-life of such proteins and greatly boosting their intra- or extracellular concentrations. This protection may reflect the mechanism by which the p54 proteasomal polyubiquitin receptor subunit prevents its own proteolytic degradation while feeding the substrates into the opened channel of the catalytic core of the 26S proteasome. The C-terminal half of subunits p54/Rpn10/S5a is intrinsically disordered [12]; therefore, its position, conformational changes and interactions with the proteasome during the major structural rearrangement accompanying the substrate feeding into the 20S core particle are not visible on cryo-electron microscopic images [21,22,23]. Therefore, the mechanism by which STABILON interferes with the proteolytic activity of the proteasomes has been elusive. Nevertheless, as revealed by our previous results, the STABILON of subunit p54 has a vital role in *Drosophila melanogaster* [5]. Deletion of the single copy *p54* gene in *Drosophila* (*Δp54*) causes polyphasic larval-pupal lethality [8], but these mutant animals die as early pupae and never reach the pharate adult stage. Transgenic expression of the *p54* gene in *Δp54* genetic background fully rescues the lethal phenotype of the deletion mutant. In contrast, transgenic expression of a *p54* gene (in *Δp54* genetic background) from which the STABILON was deleted (p54-ΔStab) only partially rescued the *Δp54*-caused lethality: the lethal phase of the *Δp54* mutation was shifted from pupa to pharate adult stage, but animals never hatched [5].

Our results reveal a hitherto unknown regulatory mechanism important for proteostasis by demonstrating that besides its protective effect preventing the fast proteasomal degradation of unstructured proteins, the conserved STABILON motif carries a *cis*-acting independent function boosting the accumulation of mRNAs of all the expressed transgenes tested in our work, independently of the structure, function, or cellular localization of the encoded proteins. Recently it has been shown that codon triplets contain translation-dependent regulatory information that influences transcript decay [24]. The abundance of stabilizing codons present in the STABILON motif may extend the half-life of the encoded mRNA. Alternatively, the binding of specific protein factor(s), which can stimulate both the accumulation of the mRNAs and the ribosomal turnover during their translation, may explain its mode(s) of action. Elaboration on this mechanism requires further research and effort.

All these findings indicate that STABILONs found at the C-termini of the polyubiquitin receptor subunit orthologues of the proteasome in higher Eukaryotes serve as dual function, *cis*-acting genuine stabilization motifs. STABILONs boost the accumulation of the mRNA of the fusion product and stabilize the short-lived, proteasomal degradation-prone proteins. This function depends on positively and negatively charged amino acids lying in their sequences in a well-defined and conserved choreography.

The production of therapeutic proteins is very expensive (up to billions of dollars per kilogram, e.g., rFVIII, EPO, rHepatitis B Surface Antigen, monoclonal antibodies, cytokines, etc. [25]) because they are often expressed in mammalian cell cultures in order to obtain proper folding, processing, activity and post-synthetic modifications [26]. Over the past years, the quality and yield of recombinant protein production in CHO cells have greatly improved. The questions remain, have we reached the biological limit of the protein production capacity of CHO cells, or can we further enhance the yield? One major issue to be solved is the (un)stability of the recombinant proteins, no matter whether it is intracellular or secreted. Our results shed light on a new level of protein homeostasis regulation and open new research avenues to improve protein production. With the application of mRNA and/or protein stabilization motifs, this problem can be overcome, and not only the production yield but the in vivo half-life (an extended effect) of the therapeutic protein could also be improved.

## 4. Materials and Methods

### 4.1. DNA Constructs

We used the pTRE2hyg vector (Clontech, Mountain View, CA, USA, cat# 6255-1) to generate recombinant plasmids suitable for the establishment of stably transfected CHO-K1 cells under the regulation of the Tet-On system. pTRE2hyg-Flag-MCS vector (for the N-terminal Flag tagging of proteins; MCS refers to the multi cloning site) was generated by cloning the 5′-ATGGATTACAAGGACGACGATGACAA-3′ Flag-tag-encoding oligonucleotide between the BamHI and PvuII sites of pTRE2hyg. pTRE2hyg-Flag-MCS-Stab vector (for the N-terminal Flag and C-terminal STABILON tagging of proteins) or pTRE2hyg-MCS-Stab (for the C-terminal STBAILON-tagging of proteins) were generated by cloning the STABILON-STOP-encoding oligonucleotide (5′-AAGGACAAGGACAAAAAGAGCGACGGCAAGGACTCGCAAAAAAAATAA-3′) PCR amplified from the cDNA of the *Drosophila* p54 [9] between the ClaI and SalI sites of the pTRE2hyg-Flag-MCS or pTRE2hyg plasmids, respectively. In the pTRE2hyg-Flag-MCS-2 × Stab and pTRE2hyg-MCS-2 × Stab plasmids, two consecutive STABILON-encoding sequences were cloned between the ClaI and SalI sites (with STOP codon downstream the second STABILON). CTH or CTH-ΔStab coding sequences were sub-cloned from the CTH/pASK-IBA5 or CTH-ΔStab/pASK-IBA5 plasmids [5] into the MluI and NheI sites of pTRE2hyg plasmid, respectively. To generate CTH-ΔStab-5 × SP2-encoding transgene, the SP2 weak initiator sequence was generated by annealing the corresponding sense and antisense oligonucleotide primers (Table S1). Five consecutive copies of SP2 were generated and cloned into the pTRE2hyg-CTH-ΔStab plasmid in frame with CTH-ΔStab followed by a STOP codon. Gly-H or ERD10 coding sequences were PCR amplified from the Gly-H/pET28 or ERD10/pET28 plasmids (kind gift from Peter Tompa [17]), and cloned into the MluI and NheI sites of pTRE2hyg-Flag-MCS or pTRE2hyg-Flag-MCS-Stab vectors, respectively, to generate Flag-Gly-H, Flag-Gly-H-Stab, Flag-ERD10 or Flag-ERD10-Stab proteins. Human erythropoietin (hEPO, Uniprot: P01588) coding sequence was PCR-amplified from the hEPO/pcDNA3.1 plasmid (kind gift from Norbert Pardi, Katalin Karikó and Drew Weissman [27]) and cloned into the MluI and NheI sites of pTRE2hyg, pTRE2hyg-MCS-Stab or pTRE2hyg-MCS-2 × Stab vectors, in frame with the STABILON sequence. To generate hEPO fused to *Drosophila* or human STABILON derivatives, hEPO was PCR amplified with hEPO fw and the appropriate STABILON derivative encoding hEPO “rev” primers and cloned into the MluI and NheI sites of pTRE2hyg plasmid. The CDS of c-Myc was PCR-amplified from mouse fibroblast first-strand cDNA library, cloned into pJET 2.1/blunt vector (Thermo Fisher Scientific, Waltham, MA, USA, cat# K1231), sequence verified and subcloned into the pTRE2hyg-Flag-MCS, pTRE2hyg-Flag-MCS-Stab or pTRE2hyg-Flag-MCS-2 × Stab plasmids, respectively, in frame with the Flag, Flag/Stab or Flag/2 × Stab sequences. DNA plasmids for fly embryo injection were made as follows: p54-CTH and CTH-ΔStab-encoding DNA were sub-cloned from CTH/pTRE2hyg or CTH-ΔStab/pTRE2hyg plasmids into pUASTattB plasmid, respectively. All DNA constructs were verified by DNA sequencing on both strands. Oligonucleotide primers are listed in Table S1.

### 4.2. Drosophila Transgenic Lines

Purified, endotoxin-free pUASTattB DNA constructs were injected into *y w M(eGFP, vas-int, dmRFP)ZH-2A*; *PattP40* embryos [11], and transformed flies were selected according to standard procedures [28]. Fly stocks were cultured at 25 °C on standard *Drosophila* food. Expression of the transgenes was induced by crossing the UAS responder lines to the Gal4 driver line (see below). Larvae, pupae and adults were collected from the vials, washed in PBS and quick frozen in liquid nitrogen. The following *Drosophila* stocks were used in this study: 

*yw M(eGFP, vas-int, dmRFP)ZH-2A; PattP40*

*w; P(pUAST-CTH); MKRS/TM6BTbHu*

*w; P(pUAST-CTH-ΔStab); MKRS/TM6BTbHu*

*w; +; P(da-Gal4).*

### 4.3. Generation of Stably Transfected Mammalian Cell Lines

Purified, endotoxin-free plasmid DNA carrying different constructs in pTRE2hyg vector or its derivatives were transfected into CHO-K1 cells (Clontech) with FuGene HD transfection reagent (Promega, Madison, WI, USA, cat# E2311) according to the manufacturer. Two days post-transfection, selection of stably transfected cell lines was started in DMEM low glucose medium (Biosera, Nuaille, France, cat# LM-D1102/500) supplemented with 10% tetracycline-free fetal bovine serum (Biosera, cat# FB-1001T) and 250 µg/mL hygromycin B (Corning, NY, USA, cat# 30-240-CR). The selection was continued for 2–3 weeks with frequent changes in the selection medium until distinct colonies could be visualized. In order to minimize the integration site-dependent differences in the expression of the transgenes, several thousand independent stable transfected colonies were pooled and maintained as a stock cell line. Expression of the transgenes was induced with 1.5 µg/mL doxycycline (Clontech, cat# 631311). Mammalian cells were kept at 37 °C humidified incubator in the presence of 5% CO_2_.

### 4.4. Cycloheximide Chase Experiment

Stably transfected CTH or CTH-ΔStab CHO-K1 cell lines were treated with 1.5 µg/mL doxycycline for 20 h (induction of transgene expression). The inducer was washed out (transcriptional blockage), and cells were further grown in a doxycycline-free medium in the presence (300 µg/mL) of the translation inhibitor drug Cycloheximide (Merck Millipore, Burlington, MA, USA, # C7698) for 0, 1, 2, 3, 4 and 5 h at 37 °C in the presence of 5% CO_2_. Cells were harvested and processed for SDS-PAGE analysis.

### 4.5. MG132 Treatment

Stably transfected CHO-K1 cell lines were treated with 0 µM (DMSO only (Sigma-Aldrich, St. Louis, MO, USA, D2438)), 2.5 µM or 5 µM MG132 (Cayman, Ann Arbor, MI, USA, cat# 10012628-10, dissolved in DMSO) for 5 h before harvesting.

### 4.6. Protein Preparation for SDS-PAGE and Mass Spectrometry

*Drosophila* larvae, pupae and adults were anesthetized and flash frozen in liquid nitrogen. Samples were homogenized in Laemmli sample buffer using glass pestles, boiled for 5 min, centrifuged at room temperature for 5 min at 17,000× *g*, and an equal amount of total protein samples (10 µg from each) were subjected to SDS-PAGE and immunoblot analysis.

hEPO-Stab-expression was induced in stably transfected CHO-K1 cells grown in Opti-MEM I reduced serum medium (Thermo Fischer Scientific, cat# 31985047) supplemented with 1.5 µg/mL doxycycline for 24 h. Cell culture supernatant was collected (10 mL) and clarified by centrifugation at 2000× *g* at room temperature for 5 min. The supernatant was precipitated with 10 *w*/*v* % of ice cold trichloracetic acid and incubated on ice for 5 min, followed by centrifugation at 17,000× *g* at 4 °C for 5 min. The precipitate was washed with ice-cold acetone, dried, resuspended in Laemmli sample buffer, boiled for 5 min and subjected to SDS-PAGE followed by Coomassie-Brilliant Blue-staining of the gels. Band corresponding to hEPO-Stab was cut and subjected to proteolytic digestion and mass spectrometric analysis.

### 4.7. Mass Spectrometry

hEPO-Stab band was cut into two pieces and subjected to in-gel digestion (at 37 °C for 4 h). Enzymes were selected to enable distinguishing processed or unprocessed versions of the proteins. hEPO-Stab was digested using trypsin or chymotrypsin, respectively. The resulting peptide mixtures were analyzed by data-dependent LC-MS/MS using an Orbitrap Fusion Lumos mass spectrometer (Thermo Fisher Scientific). Both MS1 and HCD spectra were analyzed using the Orbitrap mass analyzer. Peak lists generated from the MS/MS data by the PAVA software [29] were searched against the *Cricetulus griseus* entries of the Swissprot database (18 June 2021 version; 249 target sequences) supplemented with the sequences of the recombinant hEPO proteins using the ProteinProspector search engine (v.6.3.1.). Search parameters: enzyme: trypsin or V8 DE with maximum 1 missed cleavage; fixed modification: carbamidomethyl (Cys); variable modifications: acetylation (protein N-terminus), oxidation (Met), pyroglutamic acid formation (N-terminal Gln) allowing 2 variable modifications per peptide; mass accuracy: 5 ppm and 10 ppm for precursor and fragment ions (both monoisotopic), respectively. The following acceptance criteria were applied: score > 22 and 15, and E-value < 0.01 and 0.05 for protein and peptide identifications, respectively. Identifications assigned to peptides containing the Stab tag were inspected manually.

### 4.8. Gel-Electrophoresis and Immunoblotting

For denaturing polyacrylamide gel-electrophoresis (SDS-PAGE), we used freshly casted standard Tris-Glycine gels with stacking and separating layers. Protein gels were either stained with Coomassie Brilliant Blue R-250 (VWR Chemicals, Radnor, PA, USA, cat# M128) or blotted onto PVDF membranes (Merck Millipore, cat# IPVH00010) by semi-dry transfer (20 V, 1 h, Trans-Blot SD, Bio-Rad, Berkeley, CA, USA) in Bjerrum and Schafer-Nielsen transfer buffer (48 mM Tris, 39 mM glycine, 20% methanol and 0.037% SDS, pH ~9.2). Membranes were blocked for 30 min in 5 *w*/*v* % non-fat milk in Tris-buffered saline (TBS) and probed with the indicated antibodies for 1 h in TBS supplemented with 0.1% Tween-20 and 1 mg/mL BSA (hereafter TBST + B) at room temperature. Five-minute ECL reaction was performed using Immobilon Western Chemiluminescent HRP Substrate (Merck Millipore, cat# WBKLS0500). Membranes were exposed to X-ray films, developed in the dark following standard procedures and scanned, or chemiluminescence was detected and digitalized using Azure c300 (Azure Biosystems, Dublin, CA, USA) gel-documentation system. The following antibodies were used: mouse anti-p54 (recognizing the CTH/CTH-ΔStab region of p54, mAb SN [4,5], 1:500), mouse anti-FlagM_2_ (Sigma-Aldrich, cat# F3165, 1:10,000), mouse anti-hEPO (clone AE7A5, R&D Systems, Minneapolis, MN, USA, cat# MAB2871, 1:10,000), mouse anti-αTubulin (clone DM1α; Sigma-Aldrich, cat# T9026, 1:10,000), goat anti-mouse IgG conjugated to horseradish peroxidase (Dako, Glostrup, Denmark, cat# P044701-2, 1:10,000) and goat anti-rabbit IgG conjugated to horseradish peroxidase (Dako, cat# P044801-2, 1:10,000).

### 4.9. Densitometry Band Quantification of Western Blot Experiments

To quantify CTH and CTH-ΔStab protein levels in the cycloheximide chase experiment, digital Western blot images were subjected to densitometric analysis using the Fiji software [30] according to [31]. Normalization was done on control CHO-K1 extracts.

### 4.10. cDNA Synthesis and Quantitative Real-Time PCR

Total RNA purification from 3 × 10^6^ CHO-K1 cells was performed with a Quick-RNA MiniPrep kit (Zymo Research, Irvin, CA, USA, cat# R1054). For the synthesis of first-strand cDNA, the RevertAid First Strand cDNA Synthesis Kit (Thermo Fisher Scientific, cat# K1670) was used according to the manufacturer’s instructions. Maxima SYBR Green/ROX qPCR Master Mix (Thermo Fisher Scientific, cat# K0222) was used for the real-time quantitative PCR reaction, according to the manufacturer’s instructions. Reactions were run on three occasion times in quadruplicates in the Rotor-Gene Q Real-Time PCR Detection System (QIAGEN, USA) with the following reaction conditions: 95 °C 10 min, 40 cycles of 95 °C 15 s, 55 °C 30 s and 72 °C 30 s. The final values represent the mean and standard error of the quadruplicates. qPCR primers are listed in Table S1.

## Data Availability

Not applicable.

## Supplementary Materials

The following supporting information can be downloaded at: https://www.mdpi.com/article/10.3390/ijms23158168/s1. Reference [32] is cited in the Supplementary Figure S1.
