# Supplementary material for: STABILON, a Novel Sequence Motif That Enhances the Expression and Accumulation of Intracellular and Secreted Proteins
Source: Int J Mol Sci. 2022 Jul 25;23(15):8168. doi: 10.3390/ijms23158168 (PMC9332151; doi:10.3390/ijms23158168)

Rethi-Nagy et. al. Int. J. Mol. Sci. ***2022****, 23, 8168.*
https://doi.org/10.3390/ijms23158168

Figure S1. STABILON is conserved through evolution.
Multiple sequence alignments of the very C-terminal sequences of the proteasomal polyubiquitin receptor subunits in primates, other mammals or *Drosophilidae*. Aligned sequences were subjected to WebLogo3 [ref. 32] to generate sequence logos showing the most conserved amino acids of the various potential STABILON sequences.

Primates

```
CLUSTAL Omega(1.2.4) multiple sequence alignment


Csyr/Tarsier                      STKESKKDKKEEDKK
Ccap/Capuchin                     ATKDDKQDKKEEDKK
Ogar/Bushbaby                     ATKDGKKDKKEEDKK
Cjac/Marmoset                     ATKDGKKDKKEEDKK
Mmul/Macaque                      ATKDGKKDKKEEDKK
Hsap/Human                        ATKDGKKDKKEEDKK
Caty/Sooty_mangabey               ATKDGKKDKKEEDKK
Capa/Angola_colobus               ATKDGKKDKKEEDKK
Nleu/Gibbon                       ATKDGKKDKKEEDKK
Ccap/Capuchin                     ATKDGKKDKKEEDKK
Ggor/Gorilla                      ATKDGKKDKKEEDKK
Sbbo/Bolivian_squirrel_monk       ATKDGKKDKKEEDKK
Anan/Night_monkey                 ATKDGKKDKKEEDKK
Ppan/Bonobo                       ATKDGKKDKKEEDKK
Mnem/Pig-tailed                   ATKDGKKDKKEEDKK
Mfas/Crab-eating_macaque          ATKDGKKDKKEEDKK
Mleu/Drill                        ATKDGKKDKKEEDKKEEEHLA
Rbie/Black_snub-nosed_monk        ATKDGKKDKKEEDKK
Rrox/Golden_snub-nosed_monk       ATKDGKKDKKEEDKK
Panu/Olive_baboon                 ATKDGKKDKKEEDKK
Ptro/Chimpanzee                   ATKDGKKDKKEEDKK
Ptro/Chimpanzee                   ATKDGKKDKKEEDKK
Mmur/Mouse_lemur                  ATKDGKKDKKEEEKK
Pcoq/Coquerel's_sifaka            ATKDGKKDKKEEEKK
                                  :**:.*:*****:**
```

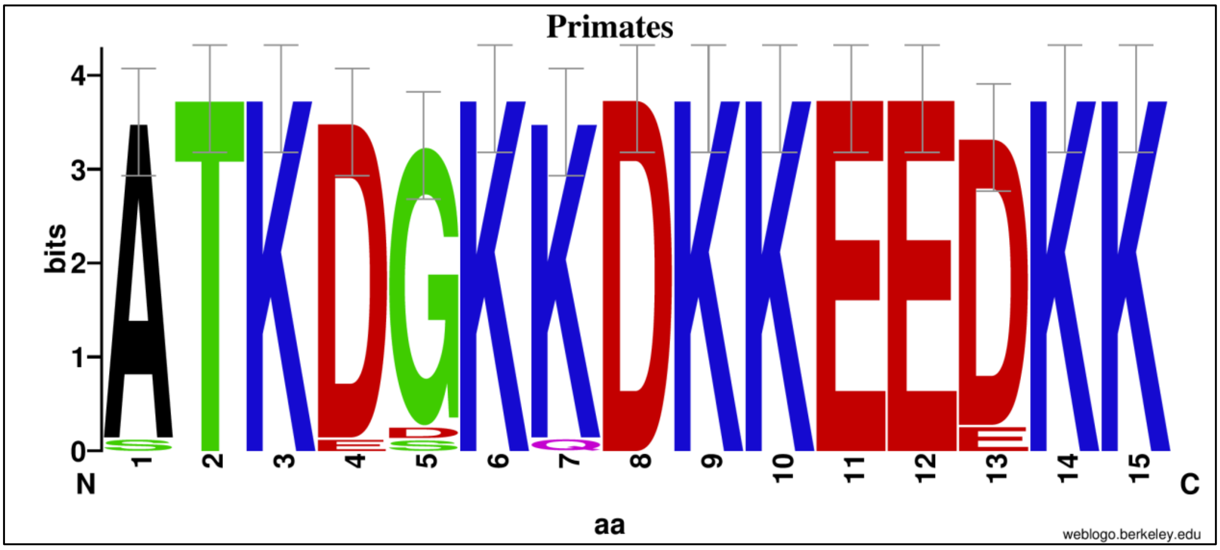

Other mammals

```
CLUSTAL Omega (1.2.4) multiple sequence alignment

Dnov/Armadillo                ATEDGKEGKKKEDKK
Lafr/Elephant                 ATKDDKQDKKEEDKK
Mdom/Opossum                  AAKGGKKEKKEEDKK
Chof/Sloth                    APKDGKKEKKEEDKK
Sara/Shrew                    GTKDGKKDKKEEDKK
Dnov/Armadillo                ATKDSKKDKKEEDKK
Pvam/Megabat                  ATKEGKKDKKEEDKK
Sscr/Pig                      ATKEGKKDKKEEDKK
Sscr/Pig                      ATKDGKKDKKEEDKK
Mluc/Microbat                 ATKDGKKEKKEEDKK
Shar/Tasmanian_devil          ATKDGKKEKKEEDKK
Neug/Wallaby                  ATKDGKKEKKEEDKK
Amel/Panda                    ANKDGKKDKKEEDKK
Mpfu/Ferret                   ANKDGKKDKKEEDKK
Fcat/Cat                      ANKDGKKDKKEEDKK
Btau/Cow                      ATKDGKKDKKEEDKKEEDKK
Ttru/Dolphin                  ATKDGKKDKKEEDKK
Tbel/Treeshrew                ATKDGKKDKKEEDKK
Opri/Pika                     ATKDGKKDKKEEDKK
Oari/Sheep                    ATKDGKKDKKEEDKK
Ocun/Rabbit                   ATKDGKKDKKEEDKK
Etel/Lesser_hedgehog_t.       ATKDGKKDKKEEDKK
Eeur/Hedgehog                 ATKDGKKDKKEEDKK
Vpac/Alpaca                   ATKDGKKDKKEEDKK
Pcap/Hyrax                    ATKDGKKDKKEEDKK
                              . : .*: **:****
```

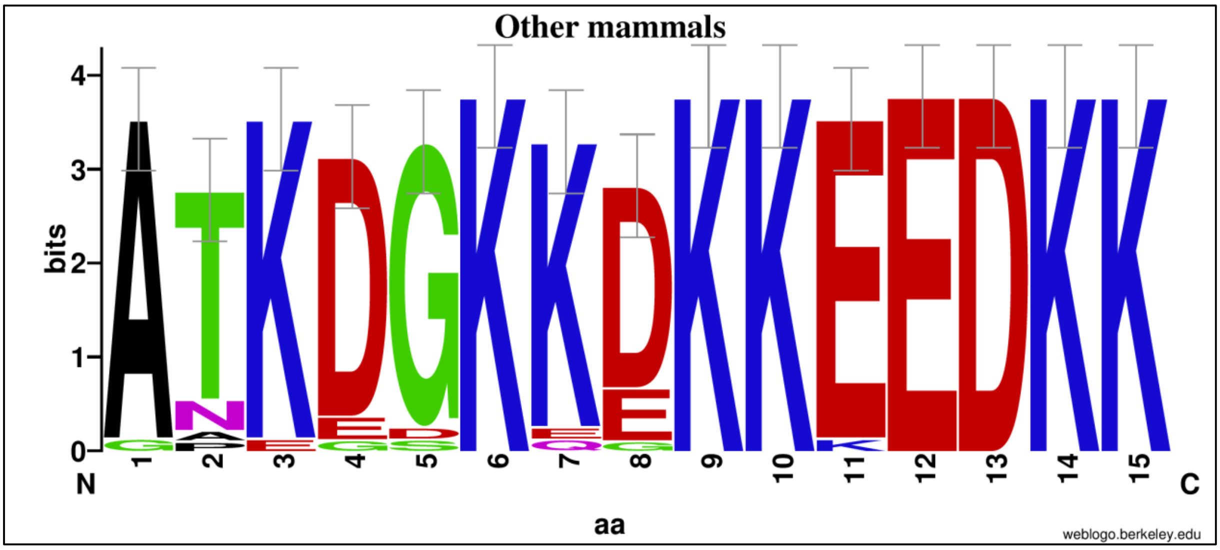

Drosophilidae

```
CLUSTAL O(1.2.4) multiple sequence alignment


Dobs       KDKDKKNEEDNQKK-
Dser       KDKDKKNDAKDSQEK
Dmir       KDKDKKNEEKDNQKK
Dpse       KDKDKKNEEKDNQKK
Dper       KDKDKKNDEKDNQKK
Dana       KDKEKKGDGKDSQKK
Dfic       KDKEKKGDGKDSQKK
Dbip       KDKEKKGDGKDSQKK
Dvir       KDKDKKNDGKDEQKK
Dhyd       KDKDKKTDGNDEQKK
Dari       KEKDKKTDGKDEQKK
Dmoj       KDKDKKTDGKDEQKK
Dbia       KDKDKKGEGKDSQKK
Dele       KDKDKKGDGKDSQKK
Drho       KDKDKKGDGKDSQKK
Dyak       KDKDKKGDGKDSQKK
Dere       KDKDKKGDGKDSQKK
Dtak       -DKDKKGDGKDSQKK
Dgri       KDKDKKSDGKDEQKK
Dmel       KDKDKKSDGKDSQKK
Dsec       KDKDKKSDGKDSQKK
Dsim       KDKDKKSDGKDSQKK
Deug       KDKDKKTDGKDSQKK
            :*:** : .:.::
```

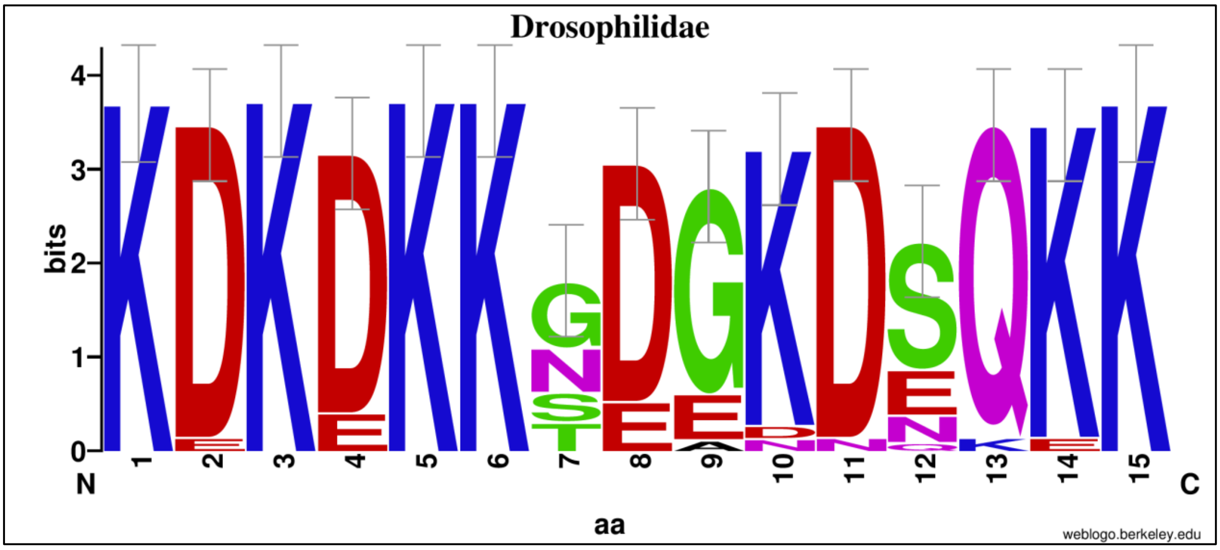

Supplement: Supplementary file 1 [file ijms-23-08168-s001.zip › Supplementary images with legends/Figure S1.pdf]
