# Supplementary material for: STABILON, a Novel Sequence Motif That Enhances the Expression and Accumulation of Intracellular and Secreted Proteins
Source: Int J Mol Sci. 2022 Jul 25;23(15):8168. doi: 10.3390/ijms23158168 (PMC9332151; doi:10.3390/ijms23158168)

Rethi-Nagy et. al. Int. J. Mol. Sci. 2022, 23, 8168.
https://doi.org/10.3390/ijms23158168

Figure S2. The half-life of p54-CTH is STABILON-dependent.
Densitrometric analysis of Western blot images (Figure 1C) shows that the *in vivo* half-life of the fruit fly CTH protein in mammalian cells is more than 5 h. In contrast, the STABILON-lacking CTH derivative (CTH-ΔStab) disappears after 4 h and its half-life is only 1 h under the same conditions.

Band intensities measured by Fiji		Hours after Cycloheximide-treatment	Band intensity percentages relative to 0h	
CTH	CTH-Δstab	h after	CTH	CTH-ΔStab
19048,48	9115,73	0	100,00	100,00
21930,36	5176,05	1	115,13	56,78
19881,77	1842,94	2	104,37	20,22
17763,70	1116,21	3	93,26	12,24
15697,53	122,12	4	82,41	1,34
11249,75	-695,07	5	59,06	-7,62

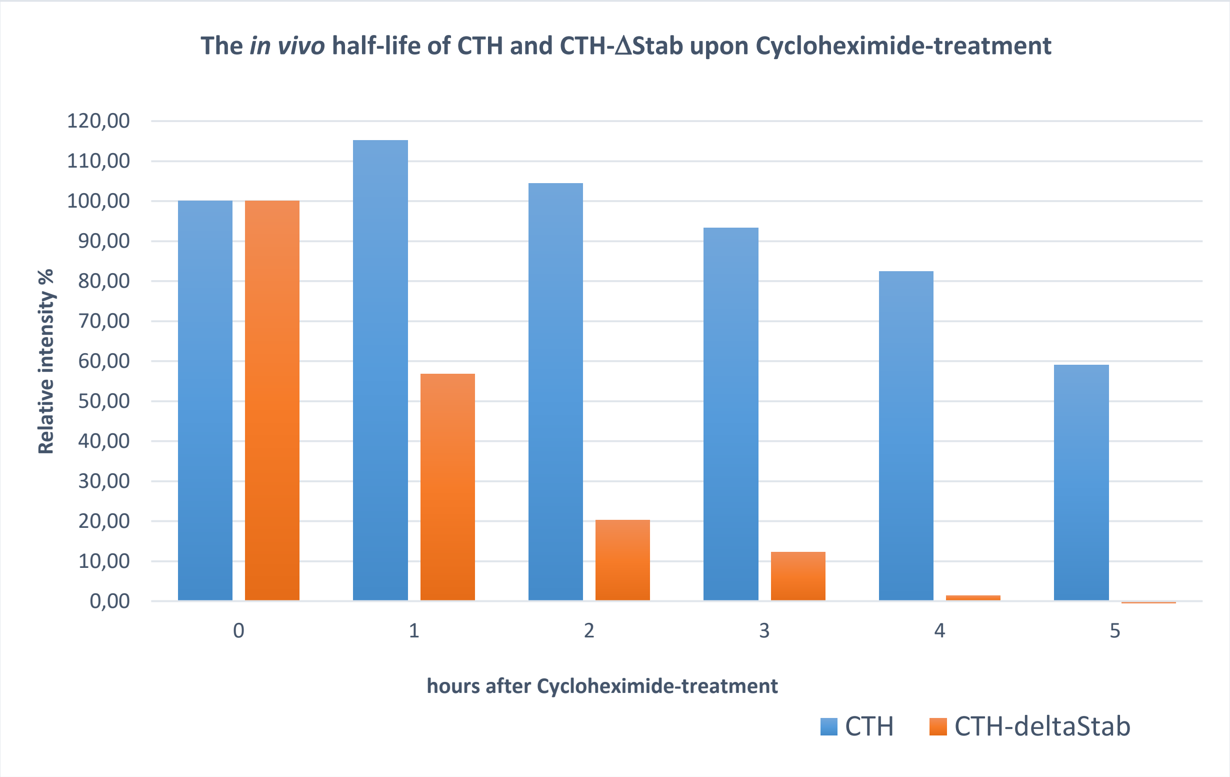

Supplement: Supplementary file 1 [file ijms-23-08168-s001.zip › Supplementary images with legends/Figure S2.pdf]
