# Supplementary material for: STABILON, a Novel Sequence Motif That Enhances the Expression and Accumulation of Intracellular and Secreted Proteins
Source: Int J Mol Sci. 2022 Jul 25;23(15):8168. doi: 10.3390/ijms23158168 (PMC9332151; doi:10.3390/ijms23158168)
Supplement: Supplementary file 1 [file ijms-23-08168-s001.zip › Supplementary images with legends/Figure S3.pdf]

Rethi-Nagy et. al. Int. J. Mol. Sci. ***2022****, 23, 8168.*
https://doi.org/10.3390/ijms23158168

Figure S3. Mass spectrometric analysis of the secreted hEPO-Stab.
Unprocessed, full-length ami-no acid sequence of hEPO-Stab is shown: the amino acid sequence of STABILON (blue) and the signal peptide of hEPO (green) are highlighted and underlined. Peptides identified by mass spectrometry after endoproteinase treatment are highlighted in red.

Signal peptide
Stabilon
hEPO sequence

hEpo-Stab protein sequence

1	MGVHECPAWL	WLLLSLLSLP	LGLPVLGAPP	RLICDSRVLE	RYLLEAKEAE	NITTGCAEHC	SLNENITVPD	TKVNFYAWKR
81	MEVGQQAVEV	WQGLALLSEA	VLRGQALLVN	SSQPWEPLQL	HVDKAVSGLR	SLTTLLRALG	AQKEAISPPD	AASAAPLRTI
161	TADTFRKLFR	VYSNFLRGKL	KLYTGEACRT	GDRASKDKKS	DGKDSQKK			

MS-identified tryptic peptides (secreted protein):

1	MGVHECPAWL	WLLLSLLSLP	LGLPVLGAPP	RLICDSRVLE	RYLLEAKEAE	NITTGCAEHC	SLNENITVPD	TKVNFYAWKR
81	MEVGQQAVEV	WQGLALLSEA	VLRGQALLVN	SSQPWEPLQL	HVDKAVSGLR	SLTTLLRALG	AQKEAISPPD	AASAAPLRTI
161	TADTFRKLFR	VYSNFLRGKL	KLYTGEACRT	GDRASKDKKS	DGKDSQKK			

MS-identified chymotryptic peptides (secreted protein):

1	MGVHECPAWL	WLLLSLLSLP	LGLPVLGAPP	RLICDSRVLE	RYLLEAKEAE	NITTGCAEHC	SLNENITVPD	TKVNFYAWKR
81	MEVGQQAVEV	WQGLALLSEA	VLRGQALLVN	SSQPWEPLQL	HVDKAVSGLR	SLTTLLRALG	AQKEAISPPD	AASAAPLRTI
161	TADTFRKLFR	VYSNFLRGKL	KLYTGEACRT	GDRASKDKKS	DGKDSQKK			
