# Supplementary material for: STABILON, a Novel Sequence Motif That Enhances the Expression and Accumulation of Intracellular and Secreted Proteins
Source: Int J Mol Sci. 2022 Jul 25;23(15):8168. doi: 10.3390/ijms23158168 (PMC9332151; doi:10.3390/ijms23158168)
Supplement: Supplementary file 1 [file ijms-23-08168-s001.zip › Supplementary images with legends/Figure S4.pdf]

https://doi.org/10.3390/ijms23158168

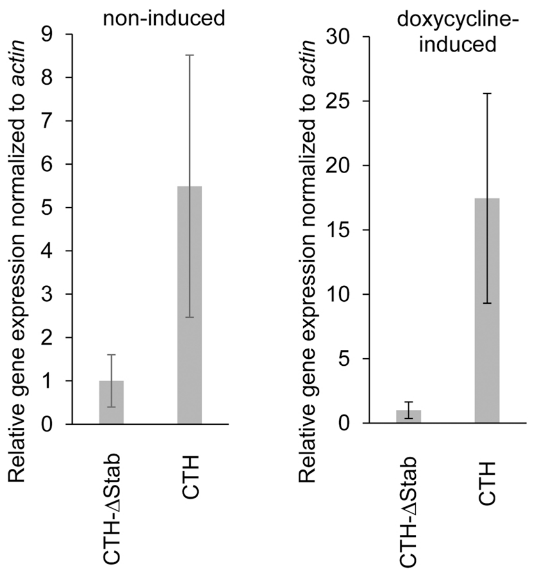

(a)

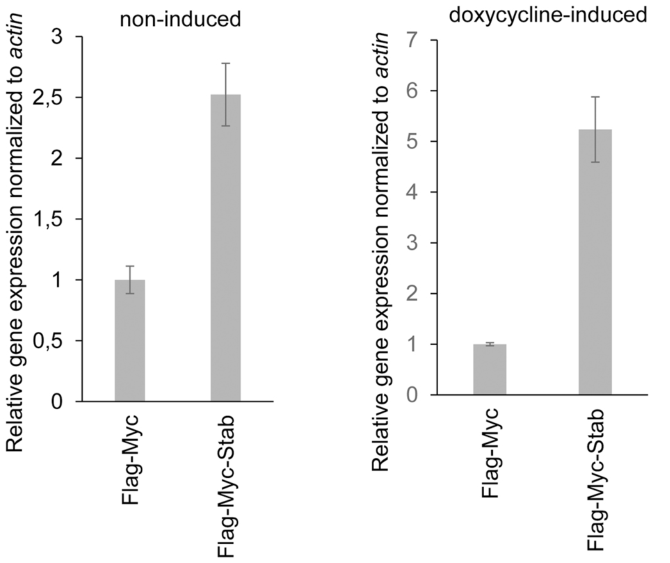

(b)

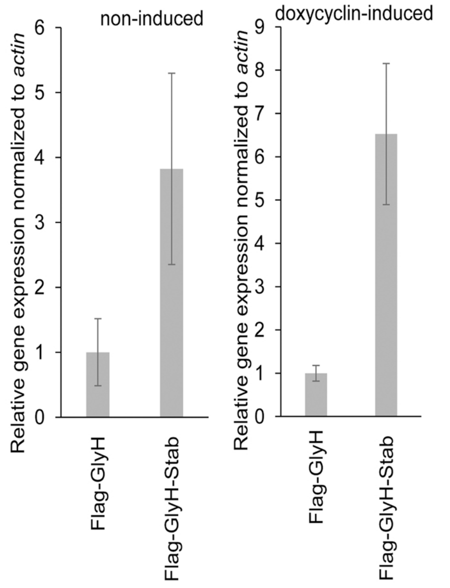

(c)

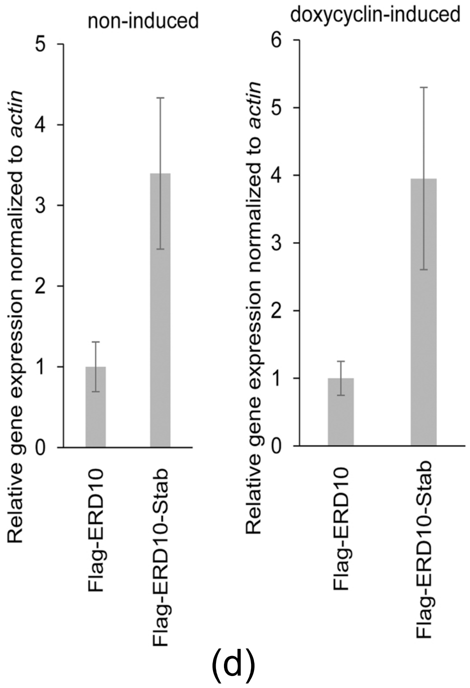

(d)

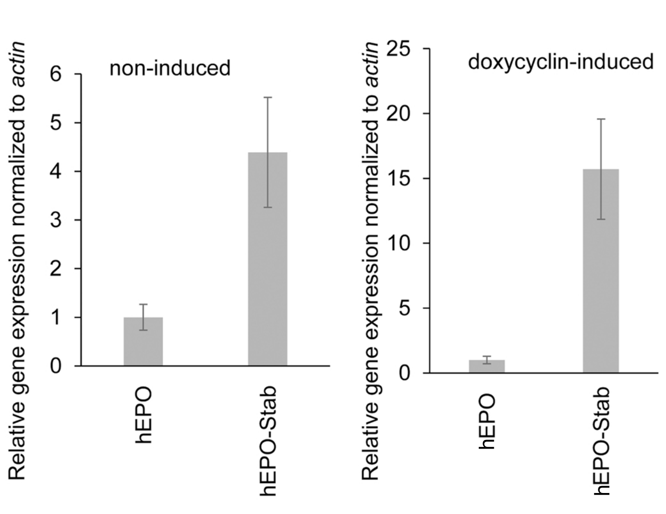

(e)

Figure S4. qRT-PCR measurements of transgene expression in CHO-K1 cells. (a) Relative tran-script levels of CTH and CTH-ΔStab transgenes in non-induced or doxycycline-induced stably transfected CHO-K1 cell lines were quantified by quantitative RT-PCR; (b) Quantitative RT-PCR analysis of Flag-tagged c-Myc or c-Myc-Stab transgenes' expression in non-induced or doxycy-cline-induced stable CHO-K1 cell lines; (c) Quantitative RT-PCR analysis of transcripts of Flag-tagged Gly-H or Gly-H-Stab transgenes in non-induced or doxycycline-induced stable CHO-K1 cell lines; (d) Quantitative RT-PCR analysis of transcripts of Flag-tagged ERD10 or ERD10-Stab transgenes in non-induced or doxycycline-induced stable CHO-K1 cell lines; (e) Quantitative RT-PCR analysis of the transcript levels of hEPO and hEPO-Stab transgenes in non-induced or doxycycline-induced stably transfected CHO-K1 cell lines. In all qPCR experi-ments the relative transcript levels were normalized to actin.
