# Supplementary material for: STABILON, a Novel Sequence Motif That Enhances the Expression and Accumulation of Intracellular and Secreted Proteins
Source: Int J Mol Sci. 2022 Jul 25;23(15):8168. doi: 10.3390/ijms23158168 (PMC9332151; doi:10.3390/ijms23158168)

Rethi-Nagy et. al. Int. J. Mol. Sci. 2022, 23, 8168.
https://doi.org/10.3390/ijms23158168

Figure S5. mRNA stability of hEPO and hEPO-Stab.
Stably transfected CHO-K1 cells expressing hEPO or hEPO-Stab proteins were induced by doxycycline treatment for 20 h. Cultures were grown for 2 h in doxycycline-free medium to allow to decline the transcription of the transgenes and further incubated in doxycycline-free medium for 0-4-7-10 hr. At the indicated time points total RNA was prepared and the hEPO or hEPO-Stab mRNA levels were measured by qRT-PCR. In all qPCR experiments the relative transcript levels were normalized to actin.

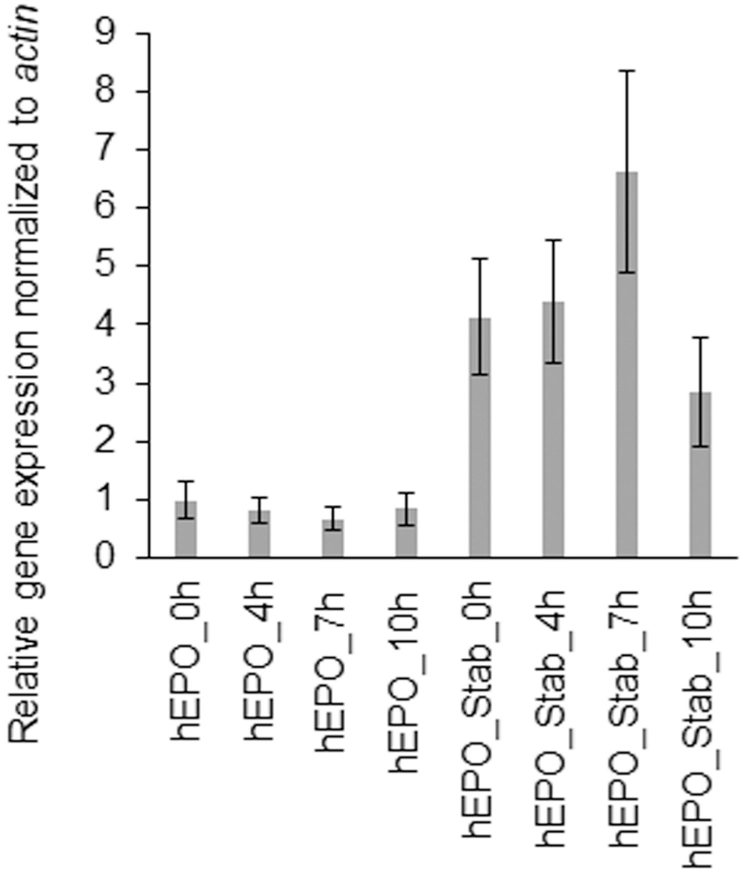

Supplement: Supplementary file 1 [file ijms-23-08168-s001.zip › Supplementary images with legends/Figure S5.pdf]
