# Supplementary material for: STABILON, a Novel Sequence Motif That Enhances the Expression and Accumulation of Intracellular and Secreted Proteins
Source: Int J Mol Sci. 2022 Jul 25;23(15):8168. doi: 10.3390/ijms23158168 (PMC9332151; doi:10.3390/ijms23158168)
Supplement: Supplementary file 1 [file ijms-23-08168-s001.zip › Supplementary images with legends/Table S1.pdf]

Rethi-Nagy et. al. Int. J. Mol. Sci. 2022, 23, 8168.
https://doi.org/10.3390/ijms23158168

Table S1. Oligonucleotide primers.
Name, sequence and application of the oligonucleotide pri-mers we used in this study. Restriction endonuclease-recognition sites are underlined.

Primer name	Primer sequence (5'-3')	Application
Gly-H fw	GTCACGCGTCCAGCAGAACTGAAATACA	Cloning into the MluI and NheI sites of pTre2hyg-Flag-MCS with the endogenous STOP codon
Gly-H rev	CAGGCTAGCGCTCGTCTTCTAACAATG	
Gly-H fw	GTCACGCGTCCAGCAGAACTGAAATACA	Cloning into the MluI and NheI sites of pTre2hyg-Flag-MCS-Stab without the endogenous STOP codon
Gly-H rev2	CAGGCTAGCCTCGTCTTCTAACAATG	
ERD10 fw	GCAACGCGTGCAGAAGAGTACAAGAACA	Cloning into the MluI and NheI sites of pTre2hyg-Flag-MCS with the endogenous STOP codon
ERD10 rev	AGCGCTAGCCATCAGACACTTTTTCTTT	
ERD10 fw	GCAACGCGTGCAGAAGAGTACAAGAACA	Cloning into the MluI and NheI sites of pTre2hyg-Flag-MCS-Stab without the endogenous STOP codon
ERD10 rev2	AGCGCTAGCATCAGACACTTTTTCTTT	
SP2 sense	CCGAGAGCATGAGGGAGGAGTACAGGAAGGAGG GCAGCAGCC	After annelaing, the 5x-SP2 sequence was cloned into the pTre2hyg-CTH-StabΔ upstream to the STOP codon.
SP2 antisense	TCGGGGCTGCTGCCCTCCTTCCTGTACTCCTCCCT CATGCTC	
c-Myc fw-jet	GATGCCCCTCAACGTGAACTTCAC	Cloning into the the pJet 1.2/blunt vector
cMyc rev-jet	GTTATGCACCAGAGTTTCGAAGCT	
c-Myc fw	CATACGCGTATGCCCCTCAACGTGAACTTCAC	Cloning into the MluI and ClaI sites of pTre2Hyg-Flag-MCS with the endogenous STOP codon
c-Myc rev	GTCATCGATTATGCACCAGAGTTTCGAAGCT	
c-Myc fw	CATACGCGTATGCCCCTCAACGTGAACTTCAC	Cloning into the MluI and ClaI sites of pTre2Hyg-Flag-MCS-Stab or pTre2hyg-Flag-MCS-2xStab without the endogenous STOP codon
c-Myc rev2	GTCATCGATTGCACCAGAGTTTCGAAGCT	
hEPO fw	GAATACGCGTATGGGGGTGCACGAATGTC	Cloning into the MluI and NheI sites of pTre2hyg to generate pTre2hyg-hEPO (with endogenous STOP)
hEPO rev	ATTAGCTAGCTCATCTGTCCCCTGTCCTGCA	
hEPO fw	GAATACGCGTATGGGGGTGCACGAATGTC	Cloning into the MluI and NheI sites of pTre2hyg-MCS-2xStab to

hEPO rev2	ATTAGCTAGCTCTGTCCCCTGTCCTGCA	generate pTre2hyg-hEPO-2xStab (without endogenous STOP)
hEPO fw	GAATACGCGTATGGGGGTGCACGAATGTC	Cloning into the MluI and NheI sites of pTre2Hyg to generate pTre2hyg-hEPO-Stab and its derivatives
hEPO-Stab$^{\text{Dm1-15}}$ rev	AATGCTAGCCTATTTTTTTTGCGAGTCTTTGCCGTCGCTTTTTTTGTCCTTGTCCTTTCTGTCCCCTGTCCTGCA	
hEPO-Stab$^{\text{Dm1-13}}$ rev	AATGCTAGCCTATTTTTTTTGCGAGTCTTTGCCGTCGCTTTTTTTGTCCTTTCTGTCCCCTGTCCTGCA	
hEPO-Stab$^{\text{Dm1-10}}$ rev	AATGCTAGCCTATTTTTTTTGCGAGTCTTTGCCGTCGCTTTTTCTGTCCCCTGTCCTGCA	
hEPO-Stab$^{\text{Dm1-9}}$ rev	CCTGCTAGCCTATTTTTTTTGCGAGTCCTTGCCGTCGCTTCTGTCCCCTGTCCTGCA	
hEPO-Stab$^{\text{Dm1-8}}$ rev	CCTGCTAGCCTATTTTTTTTGCGAGTCCTTGCCGTCTCTGTCCCCTGTCCTGCA	
hEPO-Stab$^{\text{Dm8-10}}$ rev	CCTGCTAGCCTAGTCGCTCTTTCTGTCCCCTGTCCTGCA	
hEPO-Stab$^{\text{Dm8-11}}$ rev	CCTGCTAGCCTAGTCGCTCTTTTTTCTGTCCCCTGTCCTGCA	
hEPO-Stab$^{\text{Dm8-12}}$ rev	CCTGCTAGCCTAGTCGCTCTTTTTGTCTCTGTCCCCTGTCCTGCA	
hEPO-Stab$^{\text{Dm1-7}}$ rev	CCTGCTAGCCTATTTTTTTTGCGAGTCCTTGCCTCTGTCCCCTGTCCTGCA	
hEPO-Stab$^{\text{Dm1-5}}$ rev	AATGCTAGCCTATTTTTTTTGCGAGTCTCTGTCCCCTGTCCTGCA	
hEPO-Stab$^{\text{Dm1-3}}$ rev	AATGCTAGCCTATTTTTTTTGTCTGTCCCCTGTCCTGCA	
hEPO-Stab$^{\text{Dm1-13-KR}}$ rev	AATGCTAGCCTAACGGCGTTGCGAGTCACGGCCGTCGCTACGGCGGTCGCGTCTGTCCCCTGTCCTGCA	
hEPO-Stab$^{\text{Dm1-13-KA}}$ rev	AATGCTAGCCTATGCGGCTTGCGAGTCAGCGCCGTCGCTCGCTGCGTCGGCTCTGTCCCCTGTCCTGCA	
hEPO-Stab$^{\text{Dm1-13-DA}}$ rev	AATGCTAGCCTATTTTTTTTGCGAGGCCTTGCCGGCGCTCTTTTTGGCCTTTCTGTCCCCTGTCCTGCA	
hEPO fw	GAATACGCGTATGGGGGTGCACGAATGTC	Cloning into the MluI and NheI sites of pTre2hyg to generate pTre2hyg-hEPO-Stab$^{\text{Hs1-13}}$
hEPO-Stab$^{\text{Hs1-13}}$ rev	AATGCTAGCTCACTTCTTGTCTTCCTCCTTCTTGTCCTTCTTGCCGTCCTTTCTGTCCCCTGTCCTGCA	
qPCR-actin5c_fw	ATGCCTCTGGTCGTACCACT	qPCR primers
qPCR-actin5c_rev	TCAGGTCCCGGCCAGCCA	
qPCR-CTH_fw	TCGGTGTAGATCCCAACGAGGA	
qPCR-CTH_rev	ATCTCCGCCAGTCGGTGGA	
qPCR-Flag_fw	GGACGACGATGACAAGCAGCTGAC	
qPCR-Gly-H_Rev	GCCGACGGCACTTACACCGT	

qPCR-ERD10_Rev	GCGCCGATGATTCCTCTGTTGCA
qPCR-c-Myc_rev	CTGTACGGAGTCGTAGTCGAGG
qPCR-hEPO_fw	ATGGGGGTGCACGAATGTCC
qPCR-hEPO_rev	CTCGGCTGTCACAGATGAGGCGT
